# Effectiveness of GRACE risk score in patients admitted to hospital with non-ST elevation acute coronary syndrome (UKGRIS): parallel group cluster randomised controlled trial

**DOI:** 10.1136/bmj-2022-073843

**Published:** 2023-06-14

**Authors:** Chris P Gale, Deborah D Stocken, Suleman Aktaa, Catherine Reynolds, Rachael Gilberts, David Brieger, Kathryn Carruthers, Derek P Chew, Shaun G Goodman, Catherine Fernandez, Linda D Sharples, Andrew T Yan, Keith Fox

**Affiliations:** 1 Leeds Institute of Cardiovascular and Metabolic Medicine, University of Leeds, Leeds, UK; 2 Leeds Institute for Data Analytics, University of Leeds, Leeds, UK; 3 Department of Cardiology, Leeds Teaching Hospitals NHS Trust, Leeds, UK; 4 Leeds Institute of Clinical Trials Research, University of Leeds, UK; 5 Cardiology Department, Concord Repatriation General Hospital, Sydney, Australia; 6 Centre for Cardiovascular Science, University of Edinburgh, Edinburgh, UK; 7 College of Medicine and Public Health of Medicine, Flinders University of South Australia, Adelaide, Australia; 8 Canadian VIGOUR Centre, Department of Medicine, University of Alberta, Edmonton, Canada; 9 Department of Medical Statistics, London School of Hygiene and Tropical Medicine, London, UK; 10 St Michael’s Hospital, Department of Medicine, University of Toronto, Toronto, Canada

## Abstract

**Objective:**

To determine the effectiveness of risk stratification using the Global Registry of Acute Coronary Events (GRACE) risk score (GRS) for patients presenting to hospital with suspected non-ST elevation acute coronary syndrome.

**Design:**

Parallel group cluster randomised controlled trial.

**Setting:**

Patients presenting with suspected non-ST elevation acute coronary syndrome to 42 hospitals in England between 9 March 2017 and 30 December 2019.

**Participants:**

Patients aged ≥18 years with a minimum follow-up of 12 months.

**Intervention:**

Hospitals were randomised (1:1) to patient management by standard care or according to the GRS and associated guidelines.

**Main outcome measures:**

Primary outcome measures were use of guideline recommended management and time to the composite of cardiovascular death, non-fatal myocardial infarction, new onset heart failure hospital admission, and readmission for cardiovascular event. Secondary measures included the duration of hospital stay, EQ-5D-5L (five domain, five level version of the EuroQoL index), and the composite endpoint components.

**Results:**

3050 participants (1440 GRS, 1610 standard care) were recruited in 38 UK clusters (20 GRS, 18 standard care). The mean age was 65.7 years (standard deviation 12), 69% were male, and the mean baseline GRACE scores were 119.5 (standard deviation 31.4) and 125.7 (34.4) for GRS and standard care, respectively. The uptake of guideline recommended processes was 77.3% for GRS and 75.3% for standard care (odds ratio 1.16, 95% confidence interval 0.70 to 1.92, P=0.56). The time to the first composite cardiac event was not significantly improved by the GRS (hazard ratio 0.89, 95% confidence interval 0.68 to 1.16, P=0.37). Baseline adjusted EQ-5D-5L utility at 12 months (difference −0.01, 95% confidence interval −0.06 to 0.04) and the duration of hospital admission within 12 months (mean 11.2 days, standard deviation 18 days *v* 11.8 days, 19 days) were similar for GRS and standard care.

**Conclusions:**

In adults presenting to hospital with suspected non-ST elevation acute coronary syndrome, the GRS did not improve adherence to guideline recommended management or reduce cardiovascular events at 12 months.

**Trial registration:**

ISRCTN 29731761

## Introduction

Non-ST segment elevation acute coronary syndrome (NSTEACS), which comprises non-ST elevation myocardial infarction (NSTEMI) and unstable angina, is a leading cause of disability, hospital admission, and death, and has major impacts on health economies.^1^ ^2^ NSTEACS prognosis is determined by baseline clinical risk and the use of evidence based therapies.^2^ ^3^ Although risk stratification using scores to guide the management of patients with NSTEACS is advocated in clinical guidelines, it is supported by a weak level of evidence.^4^ ^5^ ^6^

The Global Registry of Acute Coronary Events (GRACE) risk score (GRS) is designed to stratify risk in patients with acute coronary syndrome and previous research has found its discriminative performance is superior to other acute coronary syndrome risk scores.^7^ ^8^ ^9^ The Australian GRACE Risk Intervention Study (AGRIS) randomised 2318 patients with acute coronary syndrome to GRS use. Although the trial was stopped early because the intervention was found to be ineffective, implementation of the GRS was reported to be associated with an increase in early invasive treatment but no other aspects of care.^10^ Observational data for NSTEACS suggest that more comprehensive treatment is associated with improved outcomes.^11^ However, there is a lack of randomised studies that have tested whether the prospective use of the GRS improves adherence to guideline recommendations for the management of NSTEACS and reduces adverse clinical outcomes.

In the UK, hospital admission and mortality data are routinely and systematically collected. We used these data to conduct a prospective, pragmatic, cluster randomised clinical trial (UK GRS intervention study—UKGRIS) testing the hypothesis that the GRS increases guideline recommended treatment and decreases clinical events in patients admitted to hospital with suspected NSTEACS.

## Methods

### Trial design and participants

A parallel group, cluster randomised, registry based controlled trial was conducted at 42 hospitals in England. Clusters were an individual hospital or a group of hospitals that were participating in the UK national heart attack register (Myocardial Ischaemia National Audit Project). These hospitals were willing to manage suspected acute NSTEACS admissions with the intervention or standard care, and were not already using the GRS in routine clinical practice. Randomisation was at the cluster level because the GRS needed to be implemented in hospital and to avoid potential contamination among participants at the same hospital undergoing different management. The interventions were delivered at the cluster level and the outcomes measured at the participant level.

The trial design and protocol have been published elsewhere^12^ and approved by the funder, national regulatory authorities, and the ethics committee. Briefly, patients admitted to hospital with suspected NSTEACS, defined as NSTEMI or unstable angina but not ST elevation myocardial infarction, were eligible if they were aged ≥18 years, their NSTEACS was not precipitated by a clear non-cardiovascular cause, and they were not previously enrolled in the trial.

### Randomisation and masking

Eligible clusters were centrally randomised by the Clinical Trials Research Unit to the use of the GRS and suggested management or standard care (1:1). Randomisation used minimisation with a computer generated random element to ensure cluster specific volume of patients admitted to hospital with NSTEMI and primary percutaneous coronary intervention capability was similar for each study arm. When site staff were shared across hospitals, these hospitals were considered a single cluster. Site staff and participants could not be blinded.

### Intervention

All participants provided written consent before data collection. Participants recruited from hospitals randomised to the intervention arm had their GRS calculated according to European Society of Cardiology guidelines^13^ and CRUSADE (can rapid risk stratification of unstable angina patients suppress adverse outcomes with early implementation of the American College of Cardiology/American Heart Association guidelines) bleeding risk score^14^ was recorded. Hospitals randomised to standard care treated patients according to local practice and were monitored to ensure that management according to the GRS had not been systematically implemented owing to a change in hospital policy.

### Outcomes

The primary outcome measures were overall use of class I guideline recommended care processes, and time to the composite of cardiovascular death, non-fatal myocardial infarction, new onset heart failure hospital admission, or readmission for cardiovascular event within 12 months. Secondary outcome measures included the total duration of hospital stay, EQ-5D-5L (five domain, five level version of the EuroQoL index) utilities, unscheduled revascularisation, and the individual components of the composite endpoint over 12 month follow-up.

Follow-up data for use of hospital healthcare, and dates and causes of death were collected using Hospital Episode Statistics of National Health Service Digital and the Civil Registration of Deaths Register of the Office for National Statistics. These data used international classification of diseases 10th revision (ICD-10) codes.

### Sample size

We estimated that a minimum of 30 clusters, each recruiting 100 participants (3000 in total), would give 80% power to detect clinically relevant differences in the primary endpoints with two sided 5% significance tests. For the proportion of guideline directed treatments implemented, this assumed that 95% of recommended treatments would be implemented as standard based on available evidence of drug interventions at that time,^15^ and assuming uptake for cardiac imaging and rehabilitation would be similar. The trial was designed to detect an absolute increase of 3%, from 95% to 98% in the GRS arm, with an assumed coefficient of variation in cluster outcomes of 0.02.

For time to first cardiac outcome, a fixed recruitment of 15 clusters of 100 patients per arm would give 80% power to detect a reduction in 12 month event rates from 13% to 10.4% in standard care and GRS arms, respectively. These figures are based on a mean follow-up of 27 months, coefficient of variation of cluster event rates of 0.05, and loss of one cluster per arm representing up to 10% of participants.

### Statistical analyses

Analyses followed a predefined statistical analysis plan according to published guidance.^16^ Eligible guideline recommended care processes (those deemed eligible to be received) were analysed as a three level binary logistic mixed model with random intercepts for clusters and participants nested within clusters, adjusting for the cluster minimisation factors and a categorical treatment guideline identifier variable. The resulting estimate was the odds ratio for receiving a guideline directed process that a person was eligible to receive (based on their risk factors and medical history) in the population of all randomised participants irrespective of any intercurrent events such as death before discharge. Multiple imputation was used to complete information for the small number of guideline treatments for which eligibility or receipt was unknown. Analyses were based on intention to treat. Sensitivity analyses were based on complete case and single imputation (where unknown receipt was assumed not received and unknown eligibility was assumed according to a known receipt status). The statistical analysis plan prespecified a number of subgroup analyses (including interaction of intervention arm with baseline diabetes, heart failure, raised troponin, final diagnosis of NSTEACS or non-NSTEACS, GRACE and CRUSADE score categorisation, age, sex, and frailty score).

Time to first composite event and time to first of each component of the composite was analysed by Cox proportional hazards modelling, adjusted for the minimisation factors and including γ distributed random frailties. Time varying covariates were used to address any deviation from the proportional hazards assumption. Duration of hospital admission was analysed by linear regression models, adjusted for the minimisation factors with normally distributed random intercepts for hospitals. The analysis model for EQ-5D-5L utility (derived using the crosswalk algorithm) included a fixed effect for baseline EQ-5D-5L utility. Multiple imputation of incomplete or unusable EQ-5D-5L items was performed, with derived utilities overwritten with zero for participants who had died before the 12 month follow-up. All analyses were undertaken using SAS 9.4 (Cary, North Carolina, USA).

### Patient and public involvement

The UKGRIS Oversight Committee had patient representation. We did not involve patients in the interpretation of the results of the trial or the writing of the primary outcomes manuscript.

## Results

### Hospitals and patient characteristics

**Fig 1:**
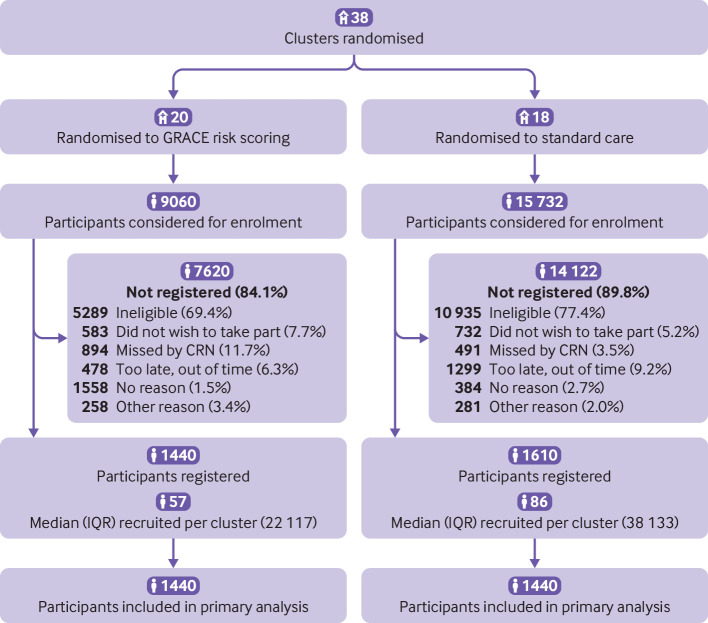
CONSORT (Consolidated Standards of Reporting Trials) diagram. CRN=clinical research network;GRACE=Global Registry of Acute Coronary Events; IQR=interquartile range

Between 9 March 2017 and 30 December 2019, we recruited 3050 participants (1440 GRS, 1610 standard care) who were admitted with suspected NSTEACS to 42 hospitals in 38 clusters (20 GRS, 18 standard care; fig 1), with mean cluster size of 80. No patients were excluded from the GRS arm or the standard care arm. Complete EQ-5D-5L data were received at baseline and 12 months for 97.8% and 75.1% of participants, respectively.

The overall mean age was 65.7 years (standard deviation 12) and 69% of patients were male (table 1). The mean GRACE scores were 119.5 (standard deviation 31.4) and 125.7 (34.4) for GRS and standard care, respectively. A low GRACE score (≤108) was recorded for 39% in the GRS arm and 32% in the standard care arm; a high GRACE score (≥141) was recorded for 24% in the GRS arm and 32% in the standard care arm. The mean CRUSADE risk score was 22.8 (standard deviation 13.4) and 23.6 (14.2) for GRS and standard care, respectively. There were 2435 (79.8%) participants with NSTEACS; 2037 (66.8%) had NSTEMI and 398 (13.0%) had unstable angina, leaving 615 (20.2%) with a final diagnosis that was not NSTEACS.

**Table 1:** Baseline characteristics of the intention-to-treat population

Characteristic	GRS cluster (n=1440)	Standard care cluster (n=1610)	Total (n=3050)
**Hospitals**			
Participants recruited per hospital, mean (SD)	65.5 (53.95)	80.5 (49.62)	72.6 (51.86)
**Volume of admissions with NSTEMI per year**			
Small (≤140)	1 (4.5)	3 (15.0)	4 (9.5)
Medium (141-270)	8 (36.4)	8 (40.0)	16 (38.1)
Large (≥271)	13 (59.1)	9 (45.0)	22 (52.4)
Hospitals with primary PCI capability	16 (72.7)	14 (70.0)	30 (71.4)
**Participant characteristics**			
Age (years), mean (SD)	65.2 (11.9)	66.2 (12.1)	65.7 (12.0)
Male sex	1015 (70.5)	1095 (68.0)	2110 (69.2)
White ethnicity, mean (SD)	1269 (88.1)	1480 (91.9)	2749 (90.1)
Body mass index, mean (SD)	29.2 (5.69)	29.4 (6.17)	29.3 (5.95)
**Medical history**			
Hypertension	646 (44.9)	849 (52.7)	1495 (49.0)
Diabetes	399 (27.7)	412 (25.6)	811 (26.6)
Peripheral vascular disease	62 (4.3)	72 (4.5)	134 (4.4)
Congestive heart failure	69 (4.8)	83 (5.2)	152 (5.0)
**Hospital admission**			
ST segment deviation on ECG	323 (22.4)	519 (32.2)	842 (27.6)
Heart rate on admission (bpm), mean (SD)	74.4 (17.09)	76.7 (18.46)	75.6 (17.86)
Systolic blood pressure on admission (mm Hg), mean (SD)	142.3 (24.75)	144.7 (24.79)	143.5 (24.80)
Cardiac arrest between symptom onset and admission	6 (0.4)	5 (0.3)	11 (0.4)
**Killip class**			
Killip I	1344 (93.3)	1426 (88.6)	2770 (90.8)
Killip II	84 (5.8)	166 (10.3)	250 (8.2)
Killip III	6 (0.4)	9 (0.6)	15 (0.5)
Killip IV	3 (0.2)	2 (0.1)	5 (0.2)
**Diuretics**	206 (14.3)	222 (13.8)	428 (14.0)
**Biomarkers**			
Creatinine (mmol/L), mean (SD)	90.0 (39.86)	91.8 (44.68)	91.0 (42.45)
Raised troponin	1175 (81.6)	1427 (88.6)	2602 (85.3)
Haematocrit (%), mean (SD)	41.1 (4.69)	41.1 (4.91)	41.1 (4.81)
**Edmonton frailty score, mean (SD)**	3.3 (2.92)	4.0 (3.26)	3.7 (3.12)
**GRACE risk score**			
Mean (SD)	119.5 (31.38)	125.7 (34.45)	122.7 (33.16)
Categories			
Unknown	6 (0.4)	32 (2.0)	38 (1.2)
Low (≤108)	562 (39.0)	520 (32.3)	1082 (35.5)
Intermediate (109-140)	524 (36.4)	535 (33.2)	1059 (34.7)
High (≥141)	348 (24.2)	523 (32.5)	871 (28.6)
**CRUSADE score**			
Mean (SD)	22.8 (13.37)	23.6 (14.23)	23.2 (13.83)
Categories			
Unknown	41 (2.8)	64 (4.0)	105 (3.4)
Low (≤30)	1016 (70.6)	1115 (69.3)	2131 (69.9)
Intermediate (31-40)	232 (16.1)	230 (14.3)	462 (15.1)
High (≥41)	151 (10.5)	201 (12.5)	352 (11.5)

Data are numbers (%) unless stated otherwise. Annualised hospital volumes of NSTEMI and primary PCI capability were abstracted from the Myocardial Ischaemia National Audit Project report covering the period 2017-20.

CRUSADE=can rapid risk stratification of unstable angina patients suppress adverse outcomes with early implementation of the American College of Cardiology/American Heart

Association guidelines; GRACE=Global Registry of Acute Coronary Events; GRS=GRACE risk score; NSTEMI=non-ST elevation myocardial infarction; PCI=percutaneous coronary intervention; SD=standard deviation.

### Processes of care

In the primary analysis, 22 473 participants were deemed eligible to receive the 11 possible guideline care processes based on GRACE category and other criteria (table 2); of these, 17 129 (76.2%) participants were confirmed to have received these care processes. Generally, the use of drug treatment if indicated was high: overall receipt of aspirin (95.4%), aspirin with P2Y_12_ inhibitor (89.5%), heparin or fondaparinux (91.1%), angiotensin converting enzyme inhibitor or angiotensin receptor blocker (82.2%), β blocker (83.4%), and statin (92.5%). By contrast, there was lower uptake of invasive angiography within 24 hours (12.2%), non-invasive ischaemia testing (24.9%) or left ventricular function assessment (59.5%), invasive angiography within 72 hours (53.7%), and cardiac rehabilitation (58.8%) among those eligible to receive these care processes.

**Table 2:** Receipt of guideline recommended care processes

Care process	GRS cluster (n=1440)	Standard care cluster (n=1610)	Total (n=3050)
Total	8121/10 505 (77.3)	9008/11 968 (75.3)	17 129/22 473 (76.2)
Aspirin	1202/1248 (96.3)	1273/1345 (94.6)	2475/2593 (95.4)
Ischaemia testing	30/110 (27.3)	12/59 (20.3)	42/169 (24.9)
Aspirin and P2Y_12_ inhibitor	799/872 (91.6)	928/1058 (87.7)	1727/1930 (89.5)
Heparin or fondaparinux	793/872 (90.9)	966/1058 (91.3)	1759/1930 (91.1)
Invasive coronary angiography within 72 h	287/508 (56.5)	266/521 (51.1)	553/1029 (53.7)
Invasive coronary angiography within 24 h	40/311 (12.9)	57/481 (11.9)	97/792 (12.2)
Left ventricular function testing	777/1440 (54.0)	1039/1610 (64.5)	1816/3050 (59.5)
ACEi/ARB	689/824 (83.6)	815/1006 (81.0)	1504/1830 (82.2)
β blockers	1205/1440 (83.7)	1338/1610 (83.1)	2543/3050 (83.4)
Statins	1345/1440 (93.4)	1475/1610 (91.6)	2820/3050 (92.5)
Cardiac rehabilitation	954/1440 (66.3)	839/1610 (52.1)	1793/3050 (58.8)

Data are number of eligible processes received divided by number of patients eligible to receive each process (%).

ACEi=angiotensin converting enzyme inhibitor; ARB=angiotensin receptor blocker; GRS=Global Registry of Acute Coronary Events risk score.

**Fig 2:**
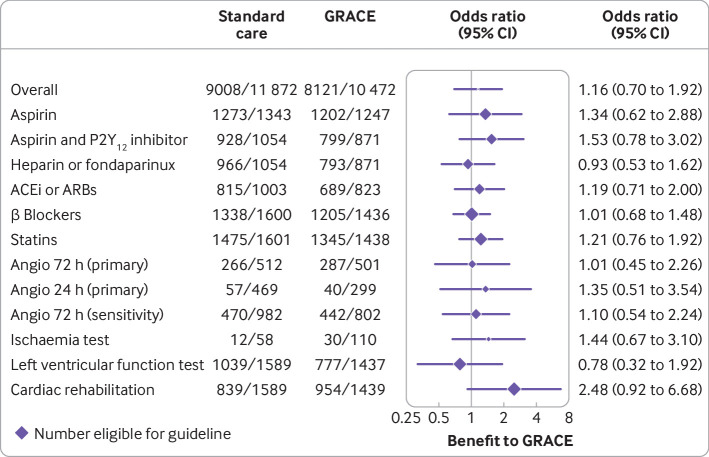
Forest plot showing effect of GRS on individual processes of care. Results were consistent in prespecified sensitivity analysis relating to definition of outcome and in an imputed dataset (odds ratio 1.19 and 1.18, respectively). ACEi=angiotensin converting enzyme inhibitor; Angio=angiography; ARB=angiotensin receptor blocker; GRACE=Global Registry of Acute Coronary Events; GRS=GRACE risk score

**Fig 3:**
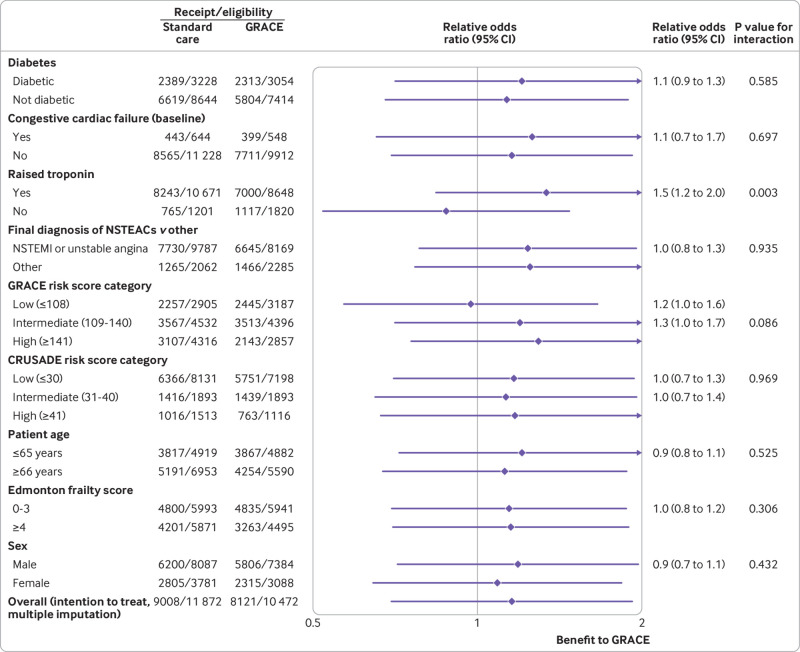
Forest plot showing baseline participant characteristics. CRUSADE=can rapid risk stratification of unstable angina patients suppress adverse outcomes with early implementation of the American College of Cardiology/American Heart Association guidelines; GRACE=Global Registry of Acute Coronary Events

Cluster randomisation to GRS did not significantly increase the rate of uptake of guideline recommended care processes compared with standard care (77.3% for GRS *v* 75.3% for standard care; odds ratio 1.16, 95% confidence interval 0.70 to 1.92, P=0.56; fig 2). Findings for planned sensitivity analyses were similar when β blockers were excluded from the process set (odds ratio 1.19, 95% confidence interval 0.69 to 2.08) or when the two invasive coronary angiography processes were combined as a single process (1.18, 0.72 to 1.93), and for sensitivity analyses involving approaches to missing data. Among GRS randomised clusters, the timing from admission to using the GRS was not significantly associated with guideline uptake (odds ratio per additional hour from admission to GRS 0.99, 95% confidence interval 0.99 to 1.00). Raised troponin on admission was associated with an increased uptake of care processes in GRS cluster randomised participants (odds ratio 1.5, 95% confidence interval 1.2 to 2.0; fig 3).

### Time to first composite cardiac event

**Fig 4:**
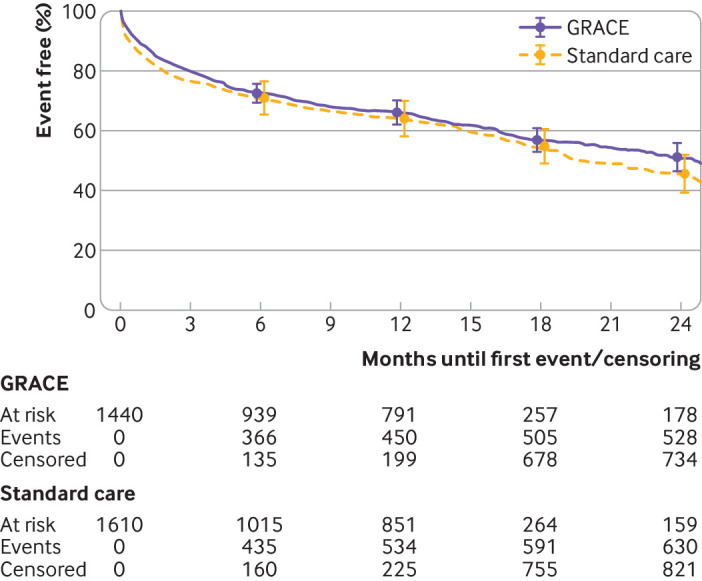
Kaplan-Meier plot of time to first clinical outcome as composite of cardiovascular death, non-fatal myocardial infarction, new onset heart failure, and cardiovascular readmission. GRACE=Global Registry of Acute Coronary Events

The overall median time to event or censoring was 12.1 months (interquartile range 1.8-13.9), with maximum follow-up at 44.5 months and similar duration of follow-up in both arms. The 12 month composite cardiac event rates were 34.0% (95% bootstrap confidence interval 29.9% to 37.8%) and 36.2% (30.2% to 42.1%) for the GRS arm and the standard care arm, respectively (hazard ratio 0.89, 95% confidence interval 0.68 to 1.16; fig 4). Sensitivity analyses adjusting for baseline GRS did not alter the results. We addressed deviation from the proportional hazards assumption by including time varying effects for the minimisation factors and this did not change the magnitude or direction of the intervention effect.

**Fig 5:**
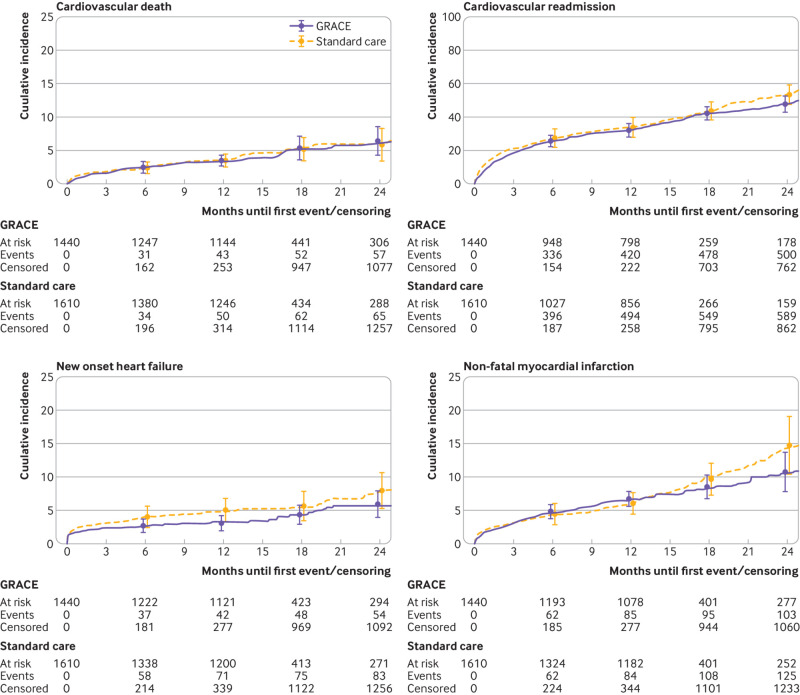
Kaplan-Meier plot of time to first clinical outcome as component parts of cardiovascular death, non-fatal myocardial infarction, new onset heart failure, and cardiovascular readmission. GRACE=Global Registry of Acute Coronary Events

The proportion of participants dying from cardiovascular causes within the first 12 months was 3.3% (95% confidence interval 2.7% to 4.1%) and 3.5% (2.4% to 4.3%) for the GRS and standard care arms, respectively (hazard ratio 1.11, 95% confidence interval 0.77 to 1.59; fig 5). The proportion of participants admitted with another non-fatal myocardial infarction or experiencing this event withing 12 months of their index admission were 6.6% (5.6% to 7.9%) and 5.9% (4.5% to 7.7%) for the GRS and standard care arms, respectively (hazard ratio 0.88, 95% confidence interval 0.68 to 1.13; fig 5). The proportion of participants admitted with new onset heart failure or experiencing this event withing 12 months of their index admission was 4.2% (2.9% to 5.7%) and 4.8% (3.3% to 6.6%) for the GRS and standard care arms, respectively (hazard ratio 0.77, 95% confidence interval 0.51 to 1.17; fig 5). The proportion of participants readmitted for cardiovascular reasons within 12 months was 32.2% (27.9% to 36.1%) and 34.1% (28.1% to 39.9%) for the GRS and standard care arms, respectively (hazard ratio 0.87, 95% confidence interval 0.67 to 1.13; fig 5).

### Quality of life

Baseline adjusted EQ-5D-5L utility at 12 months was not statistically significantly different for the two trial arms (difference −0.01, 95% confidence interval −0.06 to 0.04) with intraclass correlation coefficients ranging from 0.018 to 0.053. Duration of hospital stay within 12 months was similar (mean 11.2 days (standard deviation 18 days) and 11.8 days (19 days) for the GRS and standard care arms, respectively) and the estimated intraclass correlation coefficient was 0.015.

## Discussion

### Principal findings

In this parallel group, cluster registry based randomised clinical trial of 3050 participants admitted to hospital with suspected NSTEACS in England, the prospective use of the GRS did not improve adherence to guideline recommendations for the management of NSTEACS or reduce fatal or non-fatal cardiovascular events at 12 months compared with standard care. Additionally, baseline adjusted EQ-5D-5L utility at 12 months and the duration of hospital admission within 12 months were similar for GRS and standard care.

### Comparison with other studies

International guidelines recommend the use of the GRS to stratify patients admitted to hospital with NSTEACS so that they receive evidence based care according to their estimated risk of future ischaemic events.^4^ ^6^ Observational studies have validated the GRS across diverse populations and for a range of clinical outcomes,^17^ ^18^ and shown that a failure to follow guideline recommendations is associated with excess mortality.^19^ However, this study prospectively tested whether the GRS increases the use of guideline recommended treatments and improves clinical outcomes for people admitted to hospital with suspected NSTEACS.

The AGRIS trial found that for acute coronary syndromes including ST elevation myocardial infarction, the GRS increased the use of early invasive strategies, but not other aspects of care, for people at higher risk.^10^ In UKGRIS, we excluded patients with ST elevation myocardial infarction because in the UK these patients routinely receive an emergency invasive coronary strategy and associated drug treatments.^20^ Moreover, we sought to study the impact of the GRS on people with suspected NSTEACS as they presented to hospital, reflecting the real word situation of diagnostics and care which related to sequential investigations.^21^ Nonetheless, we did not find evidence to support the use of the GRS in increasing invasive angiography overall or in people at high risk, although compared with the use of drug treatments, the GRS did increase the referral to cardiac rehabilitation.

We found that the receipt of drug treatments was high in both arms, likely reflecting the strong evidence base for their use. Similar findings were observed in the AGRIS trial, and it is possible that the potential to show changes in care with GRS was therefore reduced. For participants with a troponin positive blood test, the GRS seemed to relate to care intervention uptake, indicating that the GRS might have an effect on patients with higher risk NSTEACS. Equally, the GRS could have a longer term impact on the delivery of care and outcomes in NSTEACS, and this will be explored in a 24 month follow-up study.

The number of coronary angiograms performed within 24 hours and 72 hours was low, possibly because physicians were uncertain about their effectiveness according to the evidence base despite recommendations for their use in international guidelines for risk stratification.^22^ ^23^ These data also suggest that participating centres might have overridden the GRS prompt. While objective risk estimation is superior to physician estimation, invasive management correlates with physician estimated risk.^24^

National routine electronic records data showed that cardiovascular events at 12 months were higher than expected. These figures were driven by a high incidence of cardiovascular hospital admissions, and less so by cardiovascular deaths, non-fatal myocardial infarctions, and new onset heart failure. The low rates of new onset heart failure were possibly because of high routine use of secondary prevention drugs.

**Strengths and limitations of this study**

We acknowledge the potential limitations of the study. Although the use of electronic health records data offers an efficient and pragmatic method by which outcomes information might be studied, we did not adjudicate the trial endpoints and it is possible that there was misclassification of events.^25^ We estimated risk according to the GRS for six month risk and applied the GRS recommendations for treatment in hospital, which might have promoted less intensive care. There was an imbalance in baseline characteristics across the two trial arms, with a higher number of participants at lower risk recruited to the GRS arm. However, we found no significant heterogeneity of the uptake in care processes by GRS categorisation, and adjusted analyses conditional on the GRS did not affect the findings (odds ratio 1.09, 95% confidence interval 0.75 to 1.58, P=0.65). Departure from anticipated estimates used in the trial design might have affected power. The coefficient of variation was in line with the expected 2% used in the sample size calculation. However, the cluster size was more variable and less than the expected 100, despite extending recruitment in smaller sites to reduce the variance. Nonetheless, the trial retained 80% power to detect the original odds ratio.

A strength of this randomised clinical trial is that all participants were followed up for admission to hospital and death using routine administrative data (including Hospital Episode Statistics and Office for National Statistics data), and the classification of their events was performed using ICD-10 codes. This strategy provides a potentially efficient mechanism through which randomised controlled trials might be delivered. Recruiting clusters were participating in the UK national heart attack register (Myocardial Ischaemia National Audit Project), and staff were therefore familiar with the study baseline variables.

### Conclusion

For participants admitted to hospital with suspected NSTEACS in England, the UKGRIS found that the GRS did not improve adherence to guideline directed management of NSTEACS or reduce cardiovascular events at 12 months compared with standard care.

**What is already known on this topic**

Adherence to guideline recommended treatments for patients with suspected non-ST elevation acute coronary syndrome (NSTEACS) improves clinical outcomes
Risk stratification using scores to guide the management of patients with NSTEACS is advocated in clinical guidelines
It is not known whether risk stratification using the Global Registry of Acute Coronary Events (GRACE) risk score (GRS) impacts the care or outcomes of patients with suspected NSTEACS

**What this study adds**

The GRS did not improve the use of guideline recommended management for NSTEASC or reduce a composite of cardiovascular death, non-fatal myocardial infarction, new onset heart failure hospital admission, and readmission for cardiovascular event
The use of the GRS compared with standard care did not reduce the duration of hospital stay or improve health related quality of life measured using EQ-5D-5L (five domain, five level version of the EuroQoL index)

## Data Availability

Anonymised centre level data might be shared upon reasonable request.

## Web extra

Extra material supplied by authors

Web appendix: Appendix

## Notes

The lead author (the manuscript’s guarantor) affirms that the manuscript is an honest, accurate, and transparent account of the study being reported; that no important aspects of the study have been omitted; and that any discrepancies from the study as planned (and, if relevant, registered) have been explained.

Dissemination to participants and related patient and public communities: Results will be shared with participants at cardiovascular meetings. A lay summary of the findings will be published on funder website and social media platforms.

Provenance and peer review: Provenance and peer review: Not commissioned; externally peer reviewed

## References

[1] Roffi M Patrono C Collet J-P ESC Scientific Document Group . 2015 ESC Guidelines for the management of acute coronary syndromes in patients presenting without persistent ST-segment elevation: Task Force for the Management of Acute Coronary Syndromes in Patients Presenting without Persistent ST-Segment Elevation of the European Society of Cardiology (ESC). Eur Heart J 2016;37:267-315. 10.1093/eurheartj/ehv320 26320110

[2] Collet JP Thiele H Barbato E ESC Scientific Document Group . 2020 ESC Guidelines for the management of acute coronary syndromes in patients presenting without persistent ST-segment elevation. Eur Heart J 2021;42:1289-367. 10.1093/eurheartj/ehaa575 32860058

[3] Fox KA Anderson FA Jr Dabbous OH GRACE investigators . Intervention in acute coronary syndromes: do patients undergo intervention on the basis of their risk characteristics? The Global Registry of Acute Coronary Events (GRACE). Heart 2007;93:177-82. 10.1136/hrt.2005.084830 16757543 PMC1861403

[4] Jneid H, Anderson JL, Wright RS, et al. 2012 ACCF/AHA Focused Update of the Guideline for the Management of Patients With Unstable Angina/Non–ST-Elevation Myocardial Infarction (Updating the 2007 Guideline and Replacing the 2011 Focused Update). *A Report of the American College of Cardiology Foundation/American Heart Association Task Force on Practice Guidelines* *.* 2012;126(7):875-910. 10.1161/CIR.0b013e318256f1e0 22800849

[5] Network SIG . Acute coronary syndromes: a national clinical guideline. (93). Scottish Intercollegiate Guidelines Network, 2007.

[6] NICE. Unstable angina and NSTEMI: the early management of unstable angina and non ST-segment-elevation myocardial infarction. (Clinical Guideline 94). 2010. 21977549

[7] Granger CB Goldberg RJ Dabbous O Global Registry of Acute Coronary Events Investigators . Predictors of hospital mortality in the global registry of acute coronary events. Arch Intern Med 2003;163:2345-53. 10.1001/archinte.163.19.2345 14581255

[8] Fox KA Dabbous OH Goldberg RJ . Prediction of risk of death and myocardial infarction in the six months after presentation with acute coronary syndrome: prospective multinational observational study (GRACE). BMJ 2006;333:1091. 10.1136/bmj.38985.646481.55 17032691 PMC1661748

[9] Gale CP Manda SOM Weston CF Birkhead JS Batin PD Hall AS . Evaluation of risk scores for risk stratification of acute coronary syndromes in the Myocardial Infarction National Audit Project (MINAP) database. Heart 2009;95:221-7. 10.1136/hrt.2008.144022 18467355

[10] Chew DP Hyun K Morton E . Objective risk assessment vs standard care for acute coronary syndromes: a randomized clinical trial. JAMA Cardiol 2021;6:304-13. 10.1001/jamacardio.2020.6314 33295965 PMC7726696

[11] Hall M Bebb OJ Dondo TB . Guideline-indicated treatments and diagnostics, GRACE risk score, and survival for non-ST elevation myocardial infarction. Eur Heart J 2018;39:3798-806. 10.1093/eurheartj/ehy517 30202849 PMC6220125

[12] Everett CC Fox KAA Reynolds C . Evaluation of the impact of the GRACE risk score on the management and outcome of patients hospitalised with non-ST elevation acute coronary syndrome in the UK: protocol of the UKGRIS cluster-randomised registry-based trial. BMJ Open 2019;9:e032165. 10.1136/bmjopen-2019-032165 31492797 PMC6731819

[13] Hamm CW Bassand JP Agewall S ESC Committee for Practice Guidelines . ESC Guidelines for the management of acute coronary syndromes in patients presenting without persistent ST-segment elevation: The Task Force for the management of acute coronary syndromes (ACS) in patients presenting without persistent ST-segment elevation of the European Society of Cardiology (ESC). Eur Heart J 2011;32:2999-3054. 10.1093/eurheartj/ehr236 21873419

[14] Subherwal S Bach RG Chen AY . Baseline risk of major bleeding in non-ST-segment-elevation myocardial infarction: the CRUSADE (Can Rapid risk stratification of Unstable angina patients Suppress ADverse outcomes with Early implementation of the ACC/AHA Guidelines) Bleeding Score. Circulation 2009;119:1873-82. 10.1161/CIRCULATIONAHA.108.828541 19332461 PMC3767035

[15] Simms AD Baxter PD Cattle BA . An assessment of composite measures of hospital performance and associated mortality for patients with acute myocardial infarction. Analysis of individual hospital performance and outcome for the National Institute for Cardiovascular Outcomes Research (NICOR). Eur Heart J Acute Cardiovasc Care 2013;2:9-18. 10.1177/2048872612469132 24062929 PMC3760578

[16] Gamble C Krishan A Stocken D . Guidelines for the content of statistical analysis plans in clinical trials. JAMA 2017;318:2337-43. 10.1001/jama.2017.18556 29260229

[17] Simms AD Reynolds S Pieper K . Evaluation of the NICE mini-GRACE risk scores for acute myocardial infarction using the Myocardial Ischaemia National Audit Project (MINAP) 2003-2009: National Institute for Cardiovascular Outcomes Research (NICOR). Heart 2013;99:35-40. 10.1136/heartjnl-2012-302632 23002253

[18] Hall M Bebb OJ Dondo TB . Guideline-indicated treatments and diagnostics, GRACE risk score, and survival for non-ST elevation myocardial infarction. Eur Heart J 2018;39:3798-806. 10.1093/eurheartj/ehy517 30202849 PMC6220125

[19] Dondo TB Hall M Timmis AD . Excess mortality and guideline-indicated care following non-ST-elevation myocardial infarction. Eur Heart J Acute Cardiovasc Care 2017;6:412-20. 10.1177/2048872616647705 27142174

[20] Dondo TB Hall M Munyombwe T . A nationwide causal mediation analysis of survival following ST-elevation myocardial infarction. Heart 2020;106:765-71. 10.1136/heartjnl-2019-315760 31732655 PMC7229897

[21] Gullick J Wu J Chew D . Objective risk assessment vs standard care for acute coronary syndromes-The Australian GRACE Risk tool Implementation Study (AGRIS): a process evaluation. BMC Health Serv Res 2022;22:380. 10.1186/s12913-022-07750-8 35317816 PMC8941820

[22] Kite TA Gershlick AH . High-risk NSTE-ACS: high time for robust data. Eur Heart J 2021;42:352. 10.1093/eurheartj/ehaa927 33188591 PMC7850134

[23] Kite TA Kurmani SA Bountziouka V . Timing of invasive strategy in non-ST-elevation acute coronary syndrome: a meta-analysis of randomized controlled trials. Eur Heart J 2022;43:3148-61. 10.1093/eurheartj/ehac213 35514079 PMC9433309

[24] Chew DP Junbo G Parsonage W Perceived Risk of Ischemic and Bleeding Events in Acute Coronary Syndrome Patients (PREDICT) Study Investigators . Perceived risk of ischemic and bleeding events in acute coronary syndromes. Circ Cardiovasc Qual Outcomes 2013;6:299-308. 10.1161/CIRCOUTCOMES.111.000072 23652735

[25] Meah MN Denvir MA Mills NL Norrie J Newby DE . Clinical endpoint adjudication. Lancet 2020;395:1878-82. 10.1016/S0140-6736(20)30635-8 32534650

