# Supplementary material for: Effectiveness of GRACE risk score in patients admitted to hospital with non-ST elevation acute coronary syndrome (UKGRIS): parallel group cluster randomised controlled trial
Source: BMJ. 2023 Jun 14;381:e073843. doi: 10.1136/bmj-2022-073843 (PMC10265221; doi:10.1136/bmj-2022-073843)
Supplement: Supplementary file 1 — Web appendix: Appendix [file galc073843.ww.pdf]

Appendix: Summary of specific guideline care processes for the co-primary outcome measure of guideline care processes, criteria for eligibility to receive and definition of receipt.

Care process	Eligibility requirement	Receipt requirement
1. Aspirin	Low or intermediate CRUSADE category	Received (any dose) during admission or prescribed (any dose) on discharge
2. Ischaemia Testing	Low GRACE category, and not undergoing angiography	Underwent one or more of cardiac MRI, radionuclide testing, stress echo or exercise tolerance testing during admission or planned post-discharge
3. Aspirin and $P2Y_{12}$ inhibitor	Intermediate or High GRACE category	Both received (any dose) during admission or both prescribed (any dose) on discharge
4. Heparin or fondaparinux	Intermediate or High GRACE category	Received (any dose) during admission
5. Angiography within 72 hours of admission*	Intermediate GRACE category	Underwent within 72 hours of admission
6. Angiography within 24 hours of admission*	High GRACE category	Underwent within 24 hours of admission
7. Non-invasive Left Ventricular Function	All participants	Underwent one or more of echo, cardiac MRI, radionuclide testing or stress echo during admission.

8.	ACE inhibition or ARB	Participants with one or more of Congestive Cardiac Failure, Diabetes and/or Hypertension	Received (any dose) during admission, or prescribed on discharge
9.	Beta blockers**	All participants	Received (any dose) during admission, or prescribed on discharge
10.	Statins	All participants	Received (any dose) during admission, or prescribed on discharge
11.	Cardiac rehabilitation	All participants	Received during admission, or prescribed on discharge

Footnotes

When determining the overall rates of eligible guidelines received, the denominator is the number of guidelines for which a participant was eligible and the numerator is the number of these that were also received. When reporting simple summary statistics, guidelines are not counted if it was not possible to determine eligibility or receipt.

* In the primary analysis, these two indices relating to invasive angiography were treated as two distinct care processes, applied to two mutually exclusive patient groups. A sensitivity analysis combined these into a single guideline, where patients of intermediate or high risk were eligible, and needed to have been received within 72 hours of admission.

** In the primary analysis, beta-blockers were included in the set of guideline-recommended care processes. They were excluded in a second sensitivity analysis.

ACE: angiotensin-converting enzyme; ARB: angiotensin-receptors blockers; GRACE: Global Registry of Acute Coronary Events
